# Uptake and effects of psychological first aid training for healthcare workers’ wellbeing in nursing homes: A UK national survey

**DOI:** 10.1371/journal.pone.0277062

**Published:** 2022-11-03

**Authors:** Mariyana Schoultz, Claire McGrogan, Michelle Beattie, Leah Macaden, Clare Carolan, Geoffrey L. Dickens

**Affiliations:** 1 School of Health and Life Sciences, Northumbria University, Newcastle, United Kingdom; 2 Centre for Health Sciences, University of the Highlands and Islands, Inverness, Scotland; Albanian University, ALBANIA

## Abstract

**Aims:**

Psychological First Aid is a brief intervention based on international guidance from the World Health Organisation. Free to access online training in the intervention was introduced during the COVID-19 pandemic in UK. We aimed to determine the uptake of Psychological First Aid training among healthcare workers in care homes in the UK and to assess its effects on their wellbeing.

**Design:**

This was a sequential mixed methods design.

**Methods:**

Healthcare workers (nurses and carers) working in care homes in the UK were surveyed about their uptake of Psychological First Aid, their stress, coping efficacy and the key concepts of Psychological First Aid (safety, calmness, hopefulness, connectedness, and accomplishment). Those that completed the Psychological First Aid training were asked to share their experiences via qualitative survey. Data collection was conducted between June and October 2021. Analyses included descriptive statistics and regression analysis. A six step thematic analysis was used to interpret the qualitative data.

**Results:**

388 participants responded to the survey. The uptake of Psychological First Aid training was 37 (9.5%). Psychological first aid was a significant predictor for coping efficacy (*β* = 17.54, *p* = .001). Participants with a physical or mental health condition experienced higher stress and lower coping regardless of PFA training. Four themes were identified from the qualitative analysis: self-awareness and growth, relationships with others, overcoming stress and accessibility.

**Conclusion:**

While this study suggests some benefits to healthcare workers in care home settings undergoing PFA the poor uptake of the training warrants further investigation.

**Impact:**

Care home staff need psychological support. This gap remains as few completed PFA training. This is the first study in UK and worldwide to look at the effects of psychological first aid on stress and coping in this population and it warrants further investigation.

## 1. Introduction

Staff working in health and social care settings in the UK have been disproportionately impacted by the COVID-19 pandemic, most acutely those individuals employed in residential nursing and care homes. During the course of the pandemic, health care workers (HCWs) in these settings have experienced dramatic changes to their working environments and workload, increases in residents’ mortality, and risk to their own health [1]. Despite the impact of COVID-19, government support was limited, further compounding the challenges faced by HCWs [2]. Nonetheless, Department of Health in England introduced free to access online training in Psychological first aid to address the challenges faced by the general workforce in UK. Thus, arose the need to evaluate how effective was the implementation of this initiative in terms of uptake and effect on the wellbeing of HCWs in care homes.

In the UK, care and residential facilities are home to approximately half a million people and the workplace of almost 1.8 million staff [3]. There is substantial evidence that care homes were disproportionately affected by the COVID-19 pandemic; 24% of deaths in UK care homes involved COVID-19 and 72% of deaths involving COVID-19 worldwide were care home residents [4]. Despite the impact of the pandemic and claims that a ‘protective bubble’ had been placed around care homes, support from the UK government for these organizations was limited and patients with COVID-19 were often transferred from hospitals to care homes to reduce pressure on services [2, 4]. In a UK-wide survey, 21% of respondents said their care home received admissions of COVID-19 positive patients being discharged from hospital, and a further 43% reported admissions of patients whose COVID-19 status was unknown [5]. In addition to increased mortality of residents, HCWs faced increased risk to their own health due to limited testing capacity and inadequate personal protective equipment in the early stages of the pandemic. Between March and July 2020, 625 deaths of health and social care workers involving the COVID-19 coronavirus were recorded in the UK [6]. It is therefore unsurprising that a number of studies have reported significant negative psychological impact of the pandemic on HCWs in nursing homes [5, 7, 8]. A systematic review of HCWs’ well-being found a high prevalence of anxiety (23.3%), depression (22.8%), and insomnia (38.8%) during the pandemic [9]. Additionally, 20% of respondents in a survey stated they had considered leaving the health care profession due to the impact of COVID-19 on their working conditions. While it is clear that the pandemic has resulted in marked negative consequences for HCWs, the challenges faced by this workforce pre-pandemic should not be understated. HCWs are likely to experience post-traumatic stress (PTSD) symptoms associated with their work [10], and PTSD among staff is associated with poorer care for patients and residents [11]. These findings highlight an urgent need for effective interventions to support the well-being of HCWs, both within and outside of the context of the pandemic.

Psychological First Aid (PFA) is an intervention designed to alleviate the impact of crisis and trauma on mental health and well-being developed by the World Health Organization [12]. However, conceptually it was first introduced in the mid-20^th^ Century with PFA models proliferating in the post 9/11 period [13, 14]. PFA was developed as a brief training course aiming to equip workers or volunteers in disaster situations to reduce victims’ initial distress, meet their psychological and physical needs, and encourage flexible coping while fostering feelings of safety, calmness, hopefulness, connectedness, and accomplishment. Unlike the now largely discredited psychological debriefing, PFA does not involve discussions about the traumatic events but focuses on providing humane and practical help. PFA can be used immediately after the traumatic event or following a hiatus. The three main principles of PFA are to look (for safety or for who needs help) listen (to those that are in distress) and link (to further support). Evidence suggests that PFA is beneficial for reducing initial effects of trauma [15] or it can be used for workers with prolonged and chronic stress. Despite the widespread implementation of PFA, and the generally unchallenged consensus around its adoption, there is surprisingly little high quality research evidence of its effectiveness. Systematic reviews have repeatedly reported a dearth of evidence [16–18]. Only recently has controlled research been conducted [19], though outcomes are knowledge and understanding of psychological support principles rather than psychological wellbeing. In part, this may have arisen from the context in which PFA is implemented, and where studies are conducted, in situations of war or natural disaster where recruitment and follow-up may be impractical. More recently, there have been increasing calls to implement PFA in ‘high risk occupational settings’ [15]. In the UK, a free-to-access online version of PFA training was introduced in June 2020 as a means of increasing the capacity of frontline staff to provide support to the public during the COVID-19 pandemic. To date the course has been signed up to by 135,000 people but to the best of our knowledge no information is available about the take up of this in the care home sector, the experiences of those completing the course, or any associated outcomes. Significantly, course objectives are not only to develop understanding of PFA, to identify to whom it is suited and how it can help them, but also to foster self- and peer- support in participants and help manage their own stress.

## 2. The study

### 2.1 Aims

This study aimed to a) identify the extent to which PFA training has been accessed by HCWs in care homes in the UK; b) compare levels of stress and coping and domains of PFA (efficacy, perceived safety, calmness, hopefulness, connectedness, and accomplishment) between HCWs in UK care homes who have and have not completed PFA training; c) gain insight into the experiences of HCWs in UK care homes who have completed PFA training.

### 2.2. Design and methods

#### 2.2.1. Setting

This study used a sequential mixed methods design. The study used online and email methods to gather data in the UK between June 2021 and October 2021. During this period Covid-19 restrictions were easing across the UK largely due to widespread vaccination, however restrictions on care home visitors and use of PPE for HCWs remained.

### 2.3. Quantitative survey

An online self-report survey was developed. Prevalence of participation in PFA training was ascertained simply by asking respondents whether they had engaged with it. Next, the response to this was used as the independent variable in analyses to determine whether completion was associated with improved outcomes relative to those who had not completed the training. Dependent variables were perceived stress and coping using validated instruments and the five potential beneficial effects of PFA highlighted in the literature, namely; efficacy, feelings of safety, calmness, hopefulness, connectedness, and accomplishment (further details below).

#### 2.3.1 Sample/participants

The survey was open to any individual working in a non-NHS care home in the UK. NHS care homes were not included due to pragmatic reasons such as being less than 20% of overall UK are homes and the time constraints related to the extra ethical approval. A purposive convenience sample was recruited from a self-selected sample of private residential and care home staff who responded to study adverts placed on social media and recruitment emails sent to UK care homes. To avoid sampling bias, study recruitment materials did not mention PFA but referred only to experiences of wellbeing during the pandemic. The sample size obtained for the survey (N = 388) exceeded the minimum recommended sample size (N = 384) to allow population estimates with a 5% confidence interval (95% confidence). The minimum sample size for the study was originally premised on a calculation to make a population estimate for PFA status. It is reasonable to ask whether the sample we recruited was sufficiently large to detect significant differences between PFA and non PFA groups. Based on previously reported scores for the PSS-14 (Mean 24.0, SD 7.7)(Cohen, 1983) we have determined that detection of a difference of 1 SD (P < .05, power 80%) would require 16 participants per group. Similarly, to detect a 1 SD difference on the CSES (Mean 159, SD = 34.7) would also require a minimum sample size of 16 per group (p < .05, 80% power) [20] (Chesney 2006).

#### 2.3.2 Data collection

Data was collected via an online survey, taking approximately 15 minutes to complete, including the following scales:

*2*.*3*.*2*.*1*. *Demographics*. An in-house demographic questionnaire was used to record participants’ age, gender, ethnicity, marital status, job title, education level, domestic care responsibilities, reported personal health status, years of experience, and their country of employment. A number of these questions allowed multiple answer responses.

*2*.*3*.*2*.*2*. *Stress*. Participants’ stress was quantified using the 14 item Perceived Stress Scale [21]. Sample Items include *‘how often have you felt you were unable to control important things in your life*?*’* and *‘how often have you felt nervous and “stressed”*?*’* were rated on a 5-point scale from 0 (*Never*) to 4 (*Very Often*) based on the extent to which the participant had felt this way in the last month. Possible scores range from 0 to 56, with higher scores indicating higher levels of perceived stress.

*2*.*3*.*2*.*3*. *Coping*. Participants’ perceived ability to cope with stress was quantified using the Coping Self-Efficacy Scale [20]. Twenty-six items including *‘Find solutions to your most difficult problems’* and *‘Sort out what can and cannot be changed’* were rated on an 11-point scale from 0 (cannot do at all) to 10 (certainly can do) based on the extent to which participants report they are able to respond to problems. Possible scores range from 0 to 286, with higher scores indicating greater coping efficacy.

*2*.*3*.*2*.*4*. *PFA outcomes*. As there is currently no existing published scale that measures the intended outcomes of psychological first aid, these concepts were quantified using a 5-item in-house scale. Participants rated the extent to which they felt *‘safe’ ‘calm’ ‘hopeful’ ‘connected to people’* and *‘able to accomplish things’* in the last month on a 5-point scale from 1 (*Never*) to 5 (*Very Often*). These concepts were identified in the literature as potentially beneficial outcomes of PFA. Items were scored individually.

### 2.4 Qualitative interviews

Participants who had reported that they had completed PFA training were invited to participate in internet-hosted focus groups, individual interviews, or free-text entry responses within an online survey. All participants opted to take part via the initial survey.

#### 2.4.1 Sample/participants

A purposive sample of all participants who reported completing PFA training in the survey and were interested to share their experiences of PFA were invited via email to take part in the qualitative element of the study to gain insight into the experiences of HCWs in UK care homes who have completed PFA training.

#### 2.4.2 Data collection

Qualitative data collection comprised of an in-house demographic questionnaire and 15 open-ended free-text entry questions adapted from the interview schedule, including items such as *“What are your thoughts/experiences about the psychological first aid training*?*”* and *“What was the most helpful aspect of PFA for you*?*”*. These questions were designed to elicit rich contextual information about care staff’s experience of PFA training in their work setting. Completion of the survey took approximately 30 minutes. The full question schedule is included in S1 File.

### 2.5 Ethical considerations

Ethical approval was granted from the Faculty of Health and Life Sciences at Northumbria University. For both phases of the study, participants were provided with an online Participant Information Sheet detailing the purpose and requirements of the studies. Informed consent was indicated using an online consent form and was required before participants could proceed with the surveys. All data were collected anonymously and stored in line with appropriate regulatory requirements.

### 2.6 Quantitative data analysis

Data were analyzed using IBM SPSS Statistics 27. Descriptive statistics were calculated to address the study aim of establishing the prevalence of PFA-training take up. Potential predictor variables were dichotomized and subject to bivariate analyses (independent samples t-tests). Predictor variables were then dummy coded; PFA group (PFA = 1, non-PFA = -1), country (England = 1, rest of UK = -1), job title (carer or senior carer = 1, nurse or manager = -1), gender (female = 1, male = -1), ethnicity (White = 1, BAME = -1), education (degree or above = 1, diploma or below = -1), marital status (married = 1, other = -1), domestic care responsibilities (care responsibility = 1, none = -1), personal health status (disability / health condition = 1, none -1), years of experience (more than 10 = 1, less than 10 = -1). Data for all outcome variables for both PFA-trained and non-PFA trained groups were inspected for normality of distribution. Skewness statistics for all were within an acceptable range indicating suitability for parametric analyses. T-tests were conducted to test for significant differences on each outcome variable for each predictor variable (Table 1). A series of multiple regressions were conducted to explore the relationship between PFA condition and each outcome, while controlling for variables significantly associated with the outcome variable in the t-test analyses.

**Table 1:** Independent samples t-test results for Phase 1.

	Stress			Coping			Safety			Calmness			Hopefulness			Connectedness			Accomplishment		
	M	SD	t	M	SD	t	M	SD	t	M	SD	t	M	SD	t	M	SD	t	M	SD	t
Job group																					
Nurse / Managers	30.04	10.42	-2.24*	116.50	70.56	4.47**	3.77	1.03	3.30*	3.14	1.06	1.42	3.02	1.05	1.88	3.52	1.05	3.68**	3.33	1.09	3.15*
(Senior) Carers	32.08	7.35		85.70	60.93		3.44	0.89		2.99	0.88		2.82	0.96		3.15	0.88		2.99	0.96	
Age Group																					
45 and over	31.09	8.72	-.87	94.10	64.73	-.85	3.56	0.99	.01	3.09	0.98	1.44	2.96	1.00	1.66	3.33	0.96	1.45	3.16	1.02	1.28
44 and under	31.88	8.32		99.99	68.08		3.56	0.89		2.95	0.88		2.79	0.98		3.18	0.95		3.02	1.00	
Gender																					
Male	28.20	9.20	-1.69	116.75	68.83	1.42	4.05	1.05	2.42*	3.20	0.95	.77	3.30	1.03	1.88	3.20	1.01	-.41	3.05	1.23	-.29
Female	31.52	8.51		95.31	65.84		3.52	0.94		3.03	0.94		2.87	0.99		3.29	0.95		3.12	1.00	
Ethnicity																					
BAME	26.80	9.14	-2.12*	161.87	58.78	4.01**	3.53	0.99	-.10	3.47	1.25	1.78	3.60	1.18	2.84*	3.73	1.22	1.90	3.60	1.12	1.92
White	31.57	8.51		93.62	64.91		3.56	0.95		3.02	0.93		2.86	0.98		3.26	0.94		3.09	1.01	
Marital Status																					
Other	32.26	8.0	2.32*	93.05	61.9	-1.09	3.52	0.9	-.85	2.99	0.9	-1.20	2.83	1.0	-1.37	3.2	0.9	-1.87	3.03	1.0	-1.79
Married	30.24	9.11		100.41	70.78		3.6	0.98		3.11	0.96		2.97	0.97		3.38	0.99		3.21	1.06	
Education																					
Diploma and below	31.83	7.93	1.74	87.31	62.73	-4.62**	3.53	0.93	-.95	3.00	0.90	-1.33	2.87	0.97	-.69	3.21	0.94	-2.21*	3.08	0.99	-.80
Degree and above	30.11	10.11		121.67	68.51		3.63	1.02		3.15	1.07		2.95	1.05		3.46	0.99		3.18	1.08	
Health Status																					
None	28.89	8.99	-6.04**	115.64	70.74	6.12**	3.72	0.93	3.57**	3.27	0.99	4.86**	3.09	1.05	3.98**	3.53	0.99	5.50**	3.31	1.08	4.06**
Disability	33.92	7.32		76.46	54.06		3.39	0.95		2.81	0.84		2.69	0.89		3.02	0.85		2.90	0.90	
Care Responsibilities																					
No	31.03	9.31	-.86	95.39	65.62	-.18	3.63	0.94	1.60	3.07	0.96	.71	2.92	1.02	.52	3.35	0.93	1.65	3.16	0.99	1.14
Yes	31.78	7.79		96.63	66.27		3.48	0.96		3.01	0.93		2.86	0.97		3.19	0.97		3.05	1.03	
Years Experience																					
Less than 10	31.59	8.48	.39	93.96	64.99	-.56	3.52	0.91	-.64	2.92	0.95	-2.14*	2.83	0.94	-1.06	3.22	0.96	-.98	3.07	1.00	-.60
More than 10	31.24	8.65		97.80	66.68		3.58	0.98		3.13	0.93		2.94	1.03		3.31	0.95		3.13	1.03	
Country																					
Rest of UK	30.36	9.16	-1.37	89.53	65.91	-1.18	3.64	0.96	.97	3.06	0.99	.24	3.00	1.01	1.26	3.31	0.98	.45	3.14	1.00	.38
England	31.73	8.35		98.56	65.91		3.53	0.95		3.03	0.93		2.85	0.99		3.26	0.95		3.10	1.02	

Stress = PSS-14, Coping = CSE

*p < 0.5,

**p < .001

### 2.7 Qualitative data analysis

Qualitative data was explored using thematic analysis, following the 6 stages proposed by Braun and Clarke [22]. First, to get familiarized with the data, two researchers (MS, CC) read all the material twice. For step two the researchers generated the initial codes. For step three the researchers searched for themes among the codes independently. The researchers then met to discuss their findings and to identify themes. For step four the researchers met and reviewed themes to determine if they corresponded to the code extracts and the overall data set. Step five the researchers defined and named the themes. Finally, before agreeing and generating key themes and sub-themes. A report (step six) on the findings was then presented to all researchers and discussed.

### 2.8 Validity and reliability/rigour

For phase one, we used PSS and CSE scales, both validated and reliable tools. Due to a lack of adequate existing tools, outcomes related to safety, calmness, hopefulness, connection, and accomplishment were based on single item scales utilized for the purpose of the current study. Given that these are the purported targets of PFA, we recommend that future studies be conducted with tools with demonstrable reliability and validity.

For phase two we considered three of the criteria to establish trustworthiness: credibility, transferability, and reliability. For credibility, we did member checking where we shared the data, interpretations, and conclusions with the participants in the qualitative part of the study. For transferability, we described the context of the study as well as the participants socio demographic data and we included a number of participants quotes. For reliability, we described the data collection and data analysis in detail. Two researchers coded the data independently and consensus was reached by team discussion.

## 3. Results/findings

### 3.1 Quantitative

Three hundred and eighty-eight participants were included in analysis of quantitative data. Of these participants, 37 (9.5%; 95% CI 6.6%– 12.5%) participants stated they had completed PFA training and 351 (90.5%, 95% CI 87.5%– 93.4%) reported that they had not. Demographic information for these groups is presented in Table 2. The demographic groups over-represented in terms of who had completed PFA training were nurses and managers as opposed to carers/senior carers, those resident in England versus those in other UK regions, and those aged over 45 years.

**Table 2:** Phase 1 demographic information.

	PFA (N = 37)		Non-PFA (N = 351)	
	N	%	N	%
**Gender**				
Female	34	91.9	332	94.6
Male	2	5.4	18	5.1
Prefer not to say	1	2.7	1	0.3
**Age group**				
16–24	3	8.1	8	2.3
25–44	16	43.2	115	32.8
45–64	18	48.6	223	63.5
65+	0	0	5	1.4
**Country**				
England	31	83.8	258	73.5
Scotland	5	13.5	59	16.8
Wales	1	2.7	18	5.1
Northern Ireland	0	0	16	4.6
**Ethnicity**				
White	33	89.2	340	96.9
Black / African / Caribbean / Black British	4	10.8	6	1.7
Asian / Asian British	0	0	2	0.6
Mixed / Multiple Ethnic groups	0	0	2	0.6
Other Ethnic group	0	0	1	0.3
**Marital Status**				
Single	12	32.4	105	29.9
Married	17	45.9	152	43.3
Separated	0	0	13	3.7
Divorced	5	13.5	38	10.8
Widowed	2	5.4	5	1.4
Other	1	2.7	38	10.8
**Health Status**				
Disability	1	2.7	9	24.5
Long-term condition	7	18.8	86	2.6
Mental health condition	6	16.2	114	32.5
None	23	62.2	170	48.4
**Domestic Care Responsibilities**				
Care for children	9	24.3	101	28.8
Care for someone with long-term condition	8	21.6	49	14
Care for someone with disability	8	21.6	32	9.1
Care for someone with mental health issues	7	18.9	42	12
None	17	45.9	173	49.3
**Job title**				
Carer	11	29.7	148	42.2
Senior Carer	6	16.2	90	25.6
Nurse	2	5.4	55	15.7
Manager	18	48.6	65	18.5
**Years of Experience**				
Less than 5	5	13.5	74	21.1
6 to 10	9	24.3	68	19.4
11 to 15	2	5.4	47	13.4
16 to 20	8	21.6	44	12.5
More than 20	13	35.1	118	33.6
**Education**				
NVQ 1 or 2	4	10.8	64	18.2
NVQ 3	6	16.2	103	29.3
Diploma	16	43.2	52	14.8
Degree	8	21,6	72	20.5
Postgraduate	2	5.4	19	5.4
Other	0	0	16	4.6
None	1	2.7	25	7.1

^1^Participants could select more than one answer for health status, domestic care responsibilities, job title, years of experience and education, meaning N exceeds 388 for these variables.

NVQ = National Vocational Qualification

Descriptive statistics are displayed in Table 3.

**Table 3:** Phase 1 descriptive statistics.

	PFA					Non-PFA				
	Mean	SD	Min	Max	α	Mean	SD	Min	Max	α
Stress	28.54	9.63	5	52	.91	31.36	8.41	2	53	.85
Coping	136.62	74.24	0	258	.96	92	63.65	0	260	.98
Safety	4.14	0.79	2	5		3.50	0.95	1	5	
Calmness	3.46	1.04	1	5		3.00	0.92	1	5	
Hopeful	3.41	1.07	1	5		2.84	0.97	1	5	
Connected	3.57	1.17	1	5		3.25	0.93	1	5	
Accomplished	3.38	1.11	1	5		3.08	1.00	1	5	

Stress = PSS-14, Coping = CSE

Table 4 displays regression analysis.

**Table 4:** Regression analysis.

Predictor	Stress			Coping			Safety			Calmness			Hopefulness			Connectedness			Accomplishment		
	*β*	*SE*	*t*	*β*	*SE*	*t*	*β*	*SE*	*t*	*β*	*SE*	*t*	*β*	*SE*	*t*	*Β*	*SE*	*t*	*β*	*SE*	*t*
PFA Group (PFA)	-1.10	.72	-1.54	17.54*	5.30	3.31	.28**	.08	3.50	.20*	.08	2.55	.24*	.08	2.89	.11	.08	1.33	.10	.09	1.14
Job (Carer)	.29	.46	.64	-4.82	3.81	-1.27	-.10*	.05	-2.06	—	—	—	—	—	—	-.11*	.06	-1.98	-.13*	.05	-2.34
Gender (Female)	—	—	—	—	—	—	-.24*	.11	-2.24	—	—	—	—	—	—	—	—	—	—	—	—
Ethnicity (White)	1.20	1.09	1.10	-19.91*	8.15	-2.44	—	—	—	—	—	—	-.26*	.13	-2.03	—	—	—	—	—	—
Education (Degree+)	—	—	—	11.63*	4.07	2.86	—	—	—	—	—	—	—	—	—	.04	.06	.62	—	—	—
Health (Disability)	2.28**	.43	5.35	-16.02**	3.14	-5.10	-.14*	.05	-2.94	-.22**	.05	-4.64	-.17**	.05	-3.48	-.23**	.05	-4.88	-.18**	.05	-3.49
Experience (>10 years)	—	—	—	—	—	—	—	—	—	.01*	.05	2.04	—	—	—	—	—	—	—	—	—
Marital Status (Married)	-.69	.43	-1.62	—	—	—	—	—	—	—	—	—	—	—	—	—	—	—	—	—	—

*p < 0.5,

**p < .001

#### 3.1.1 Stress

Reported personal health status was the only significant positive predictor of stress (*β* = 2.28, *p* < .001), as participants with a disability or physical or mental health condition (*M* = 34.01, *SD* = 7.24) reported significantly higher perceived stress than those with no health conditions (*M* = 28.83, *SD* = 9.00).

#### 3.1.2 Coping

PFA was a significant positive predictor of coping (*β* = 17.54, *p* = .001), suggesting PFA training is associated with greater coping efficacy (PFA *M* = 136.62, *SD* = 74.24, Non-PFA *M* = 92, *SD* = 63.65). Ethnicity significantly predicted coping (*β* = -19.91, *p* = .02), with white participants (*M* = 93.62, *SD* = 64.91) reporting lower coping efficacy than the BAME respondents (*M* = 161.87, *SD* = 58.78). Education was also a significant predictor (*β* = 11.63, *p* = .01), participants with a degree or higher qualification (*M* = 121.67, *SD* = 68.51) reported greater coping efficacy than those with a diploma or lower qualification (*M* = 87.31, *SD* = 62.73). Finally, personal health status was a significant predictor of coping (*β* = -16.02, *p* < .001), participants with a disability or health condition (*M* = 76.46, *SD* = 54.06) scored lower for coping efficacy than those without (*M* = 115.64, *SD* = 70.74).

#### 3.1.3 Safety

Feelings of safety were positively associated with PFA training (*β* = 0.28, *p* < .001; PFA *M* = 4.14, *SD* = .79, Non-PFA *M* = 3.50, *SD* = .95). Job title was a significant negative predictor (*β* = -.10, *p* = .04), with carers and senior carers (*M* = 3.44, *SD* = .89) reporting significantly lower levels of perceived safety than nurses and managers (*M* = 3.77, *SD* = 1.03). Females (M = 3.52, SD = .94) were also found to report significantly lower perceived safety than males (*M* = 4.05, *SD* = 1.05), *β* = -.24, *p* = .03. Participants with a disability or health condition (*M* = 3.38, *SD* = .95) reported lower perceived safety than those without (*M* = 3.73, *SD* = .92), *β* = -.14, *p* = .004.

#### 3.1.4 Calmness

PFA was a significant positive predictor of feelings of calmness (*β* = .20, *p* = .01), with participants who had completed PFA training (*M* = 3.46, *SD* = 1.04) reporting significantly higher perceived calmness than those in the non-PFA group (*M* = 3, *SD* = .92). Participants with more than 10 years’ experience in healthcare (*M* = 3.12, *SD* = .93) reported significantly higher perceived calmness than those with less than 10 years’ experience (*M* = 2,92, *SD* = .91), *β* = .01, *p* = .04. Participants with a disability or health condition (*M* = 2.81, *SD* = .83) reported significantly lower perceived calmness than those without (*M* = 3.27, *SD* = .99), *β* = -.22, *p* < .001.

#### 3.1.5 Hopefulness

PFA was a significant positive predictor of hopefulness (*β* = .20, *p* = .004), with participants who had completed PFA training (M = 3.41, SD = 1.07) reporting significantly higher perceived hopefulness than those in the non-PFA group (*M* = 2.84, *SD* = .97). Ethnicity was a significant negative predictor (*β* = -.26, *p* = .04), with white participants (*M* = 2.86, *SD* = 98) reporting significantly lower perceived hopefulness than BAME participants (*M* = 3.60, *SD* = 1.18). Participants with a disability or health condition (*M* = 2.69, *SD* = .89) also reported lower hopefulness than those without (*M* =, 3.09 *SD* = 1.05), *β* = -.17, *p* < .001.

#### 3.1.6 Connectedness

Job title was a significant negative predictor of connectedness (*β* = -.11, *p* = .05), with carers and senior carers (*M* = 3.15, *SD* = .88) reporting significantly lower levels of perceived connectedness than nurses and managers (*M* = 3.52, *SD* = 1.05). Personal health status was also a significant negative predictor, with participants with a disability or health condition (*M* = 3.02, *SD* = .84) reporting lower perceived connectedness than those without (*M* = 3.53, *SD* = .98), *β* = -.23, *p* = < .001.

#### 3.1.7 Accomplishment

Job title was a significant negative predictor of feelings of accomplishment (*β* = -.13, *p* = .02), with carers and senior carers (*M* = 2.99, *SD* = .96) reporting significantly lower levels of perceived ability to accomplish things than nurses and managers (*M* = 3.33, *SD* = 1.09). Personal health status was also a significant negative predictor, with participants with a disability or health condition (*M* = 2.90, *SD* = .90) reporting lower perceived connectedness than those without (*M* = 3.31, *SD* = 1.08), *β* = -.18, *p* < .001.

### 3.2 Qualitative findings

Of the 37 participants contacted, 9 accessed the qualitative survey. 3 participants were removed from the dataset as their responses were incomplete (they only completed demographics), six participants provided sufficient data for analysis.

Demographic information for phase 2 is displayed in Table 5.

**Table 5:** Phase 2 demographic Information.

	N	%
**Gender**		
Female	6	100
**Age group**		
25–44	3	50
45–64	3	50
**Country**		
England	4	66.7
Scotland	1	16.7
Wales	1	16.7
**Ethnicity**		
White	4	66.7
Black / African / Caribbean / Black British	1	16.7
Asian / Asian British	1	16.7
**Marital Status**		
Married	4	66.7
Widowed	1	16.7
Other	1	16.7
**Health Status**		
Long term condition	1	16.7
None	5	83.3
**Domestic Care Responsibilities**		
Care for children	3	50
None	3	50
**Years of Experience**		
Less than 5	1	16.7
6 to 10	1	16.7
16 to 20	1	16.7
20 +	3	50
**Job title**		
Carer	1	16.7
Senior Carer	1	16.7
Nurse	2	33.3
Manager	2	33.3
**Education**		
NVQ 3	2	33.3
Degree	2	33.3
Postgraduate	2	33.3

Four themes were generated from data analysis: i) Self-awareness and growth, ii) Relationships with others, iii) Overcoming stress and iv) Accessibility.

#### 3.2.1 Self-awareness and growth

After PFA training, participants reported becoming ‘*more mindful’ (participant 1)* and self-aware enabling them to recognize when they were at their limits, to be confident to state this to others, and to know when to seek help. One participant described how such enhanced self-awareness was also facilitated recursively from colleagues who had also received PFA training

“*Training allowed myself to notice my own limits and seek advice when I felt I was heading for burn out*.”

*(Participant 2)*

“*A colleague supported me using her knowledge of the training*. *I found the support useful as I was able to take a step back when I found myself worrying more*.”

*(Participant 6)*

Others reported how training changed their perspective, increased their confidence, and enabled them to adopt a more relaxed approach and reported PFA to be helpful both in their work and home lives and enabling them to become a ‘*better person’ (participant 1)*

“*After training I now look at everything with a different perspective more relaxed and supportive to others*.”

*(Participant 3)*

“*Helps look at the day and organize my workload and whom I can share information or ask for help from*.”

*(Participant 3)*

“*For me it helped me function better at home and look after my children*.*”*

*(Participant 6)*

#### 3.2.2 Relationships with others

Participants described that PFA improved their relationships with others by helping them create new relationships with those around them and promoted mutual bonding. For some participants this happened through shared understanding of others in similar situations, or by having others help them make sense of their own experiences.

“*It has helped us to make new relationships and to bond with people around; PFA training increased the understanding of others in the similar situations and the measures to overcome*.”

*(Participant 4)*

“*Just having someone listen and letting me make sense of my experiences*.”

*(Participant 6)*

Some participants described that an enhanced perspective and shared understanding with others, had strengthened teamwork by helping them to “*understand workload and other peoples’ pressures*” (*Participant 3*). Some described how PFA had improved their relationships with residents, residents’ families and visitors as well and in their personal lives PFA had “*made it easier to help friends and family” (participant 1)*

“*I think I it helped teamwork as it makes staff more aware of PFA*.”

*(Participant 2)*

“*More understanding how the pandemic has hindered us as workers but also from the perspective of visitors/family and residents especially those with Dementia*.”

*(Participant 3)*

#### 3.2.3 Overcoming stress

Participants explained that the PFA training helped them separate their work and home stresses and thus enabled them to better deal with the stress caused by the increased pressures and longer working hours as well as the worries about their families.

“*Helped me to separate work from home life as I do not take the stress home or work—this is good*.”

*(Participant 3)*

Some participants described that it helped them cope better when thinking of giving up [? their job] and fostered resilience. Others described how it helped support them in their experiences of bereavement to overcome the trauma of the pandemic.

“*Has helped me cope better*, *it was a position I was thinking of giving up at one time now I have the strength to carry on*”

*(Participant 3)*

“*I found it useful as it helped me cope with bereavement as well as the experience of seeing relatives affected by COVID-19*.”

*(Participant 6)*

“*It can be really helpful to recover the trauma*.”

*(Participant 4)*

One participant highlighted how they believe PFA may have universal applications in helping overcome distress.

“*I think it is useful for anyone suffering mental distress despite profession or grade*.”

*(Participant 6)*

#### 3.2.4 Accessibility

When asked about how easy it was to access or complete the training, participants reported that there were difficulties in accessing PFA. The training was not readily available at work, they sometimes had to find out about the training via other people or search for it themselves and often there was little in-house support for it.

“*This was not something that was part of my organizations well-being support however I had a friend who had trained in it who supported me during times of crisis during the pandemic*”

*(Participant 6)*

“*I received some support with regards training*, *but the support could have been better*. *Any resources I had to search for myself*.”

*(Participant 2)*

However, once they accessed the training, it provided a ‘good way’ to challenge oneself with suggestions from a couple of participants that this training should be made mandatory for care staff during the pandemic and beyond.

“*It was a good level made me think and challenged my thoughts in a good way*.”

*(Participant 3)*

“*Should be made compulsory for all staff especially in nursing an care homes during the pandemic or not*.”

*(Participant 2)*

## 4. Discussion

The key aims of the current study were to ascertain the extent to which PFA training had been accessed by individuals working in the private care home sector in the UK and to make a first evaluation of the impact PFA had on their stress and coping. Our study found a poor uptake of PFA training 9.5% (95% CI 6.6%– 12.5%). Care home staff have a long history of being overlooked in terms of access to professional development and training. Historically, there was no current professional development pathway for HCWs in care homes. However, this has improved in recent years with the advent of HCWs registration and requirement to work through vocational qualifications in Scotland [23] and mandatory training requirements across the UK [19, 24, 25]. The reason for the poor uptake requires further investigation but it is likely to be multi-factorial including both individual worker, care home infrastructure and organisational challenges.

Sample characteristics were compared with earlier survey such as the King’s Fund [26]. However, of n = 37 people who had completed PFA training there was very little representation of males (5.4%); indeed, males appear to be under-represented in our survey in relation to their presence in the health and social care workforce (20%). BAME individuals (10.8%) also comprised a smaller than expected proportion of respondents compared to their presence in the health and social care workforce (20%). Additionally, respondents identifying themselves as managerial (48.6%) were over-represented compared to 11.1% [26] and unqualified workers were under-represented (45.9% compared with 67% in the King’s Fund report). Additionally, in the current sample a significantly larger proportion of registered nurses and manager participants had completed PFA training than unregistered carers. While the PFA operational model expects that the training will result in improved outcomes for the wider staff workforce who have contact with those who engage in it, it is also anticipated that participants will benefit personally from learning to better manage their own stress. On the current evidence, unregistered staff have had less opportunity to engage in PFA training and this has implications for future provision including consideration of the suitability of the training for this group, and whether they are able to access it in working time. Additionally, a smaller proportion of non-England based respondents had completed the training; this however could well be explained by the origins of the current roll-out as an initiative by Public Health England.

We examined outcomes related to stress, coping, safety, calmness, hopefulness, connectedness, and accomplishment in relation to PFA training status while controlling for covariates. There were significant differences between PFA and non-PFA groups on coping, safety, calmness, and hopefulness but not on stress, connectedness, or accomplishment. In all cases where a difference was present, those in the PFA group responded more favourably than those who had not participated in training. However, the effect size achieved for the relatively enhanced coping score in the PFA group was small (d = 0.27) both in itself and in the context of effect sizes achieved by psychological interventions more broadly [27]. Further, higher stress scores were associated with having a disability or long-term health condition. Coping was associated with lower scores among white participants but, given the underrepresentation of BAME individuals detailed above, it is unlikely that this result would be adversely affected by a more balanced profile of participants. Coping was also associated with higher scores among those with a higher level of education. It is worth noting that while education between the groups were similar, the proportion of those with degree or higher was higher than the average for this population [26]. Of the remaining outcomes, greater calmness was associated with not having a disability or long-term health condition and having more than 10 years’ experience. Increased safety was associated with higher job status, male gender and not having a disability. This is perhaps unsurprising given the greater proportion of time that care workers spend in ‘hands on’ roles with residents. Finally, hopefulness was associated with BAME identity and not having a disability. Connectedness and accomplishment were both associated with higher job status and not having a disability. From the data, we can conclude that, there is little to suggest that PFA training is likely to be linked to adverse outcomes. This is important information going forward. Second, the presence of increased coping in those who had received PFA gives an indication that it is possible that this was, in some part, an effect of the PFA training. Of course, in a study using an ex-post facto design with a self-selecting sample, we can only have limited confidence. However, it strongly supports the case for further investigation of the effectiveness of PFA for coping-related outcomes and for further exploratory work into the mechanisms of any effects. Given the apparently low penetration of PFA training in the care home sector these results suggest there is considerable scope for its further take-up to contribute to care worker wellness.

Qualitative results suggest participants find PFA training useful for overcoming stress, improving self-awareness and personal growth, and improving relationships with others. These findings support those of the quantitative phase of the study to an extent, suggesting PFA is useful for this population with little indication of potential to cause harm. Concerns were raised by participants over limited accessibility of PFA training which is also in line with the low penetration rates found in the quantitative survey.

When triangulating the data from both sequences, there is an indication that the qualitative data supports the quantitative data. The issues around accessibility possibly explain the low uptake of the intervention in this population. Overcoming stress as a theme supports the trends of stress and coping outcomes in PSS and CSE and provides tentative insight into mechanisms for this. CSE scale conceptualizes that social network as well as self-awareness and being able to stop being unpleasant emotions and thoughts are important aspects of coping and these are supported by the themes of relationships with others and personal growth.

### 4.1 Limitations

This was a retrospective study using a self-selected sample and significant outcomes can only be described as associations. The sample appears not to be wholly representative of the demographics of the wider care home worker population; however, there was little to suggest that results would be different among a more representative sample. Results should be interpreted with caution, as although the outcomes of interest were theoretically informed, no formal tests to establish validity and reliability were conducted and there are known limitations to measuring social constructs using single item scales.

## 5. Conclusion

While this study suggests some benefits to healthcare workers in care home settings undergoing PFA, the poor uptake of the training warrants further investigation.

### 5.1 Future research

Given that five outcome constructs (safety, calmness, hope, connection, and accomplishment) are the purported targets of PFA then we recommend that future studies be conducted with tools with demonstrable reliability and validity. These may need to be developed and designed as part of a future programme of research. Little has been written about the development of PFA and, at present, there is little evidence about how it has been operationalised. In particular whether the target audience has had adequate input into the co-production of PFA training and research. We therefore recommend that consideration be given to funding an integrated programme of research and development to further develop, implement, and evaluate a co-produced iteration of PFA for use in the UK care home sector and beyond. Elements should include national survey, co-production workshops, outcomes tools development, pilot trial, full trial, economic evaluation.

## Supporting information

S1 File (DOCX)

## References

[1] McMichael Temet M., Currie Dustin W., Clark Shauna, Pogosjans Sargis, Kay Meagan, Schwartz Noah G., Lewis James, et al. 2020. “Epidemiology of Covid-19 in a Long-Term Care Facility in King County, Washington.” New England Journal of Medicine 382 (21): 2005–11. doi: 10.1056/NEJMoa2005412 32220208 PMC7121761

[2] Rajan Selina, Adelina Comas-Herrera, and Martin McKee. 2020. “Did the UK Government Really Throw a Protective Ring around Care Homes in the COVID-19 Pandemic?” Journal of Long Term Care, November, 185–95. doi: 10.31389/JLTC.53

[3] “Care Home Stats: Number of Settings, Population & Workforce—Carehome.Co.Uk Advice.” n.d. Accessed November 25, 2021. https://www.carehome.co.uk/advice/care-home-stats-number-of-settings-population-workforce.

[4] Comas-Herrera, Adelina, Joseba Zalakaín, Elizabeth Lemmon, David Henderson, Charles Litwin, Amy T Hsu, et al. 2020. “Mortality Associated with COVID-19 in Care Homes: International Evidence.” https://www.ecdc.europa.eu/sites/default/files/documents/Increase-fatal-cases-of-COVID-19-.

[5] The Queen’s Nursing Institute. 2020. “The Experience of Care Home Staff During Covid-19.” https://www.qni.org.uk/wp-content/uploads/2020/08/The-Experience-of-Care-Home-Staff-During-Covid-19-2.pdf.

[6] “Deaths Involving COVID-19 in the Care Sector, England and Wales—Office for National Statistics.” n.d. Accessed November 25, 2021. https://www.ons.gov.uk/peoplepopulationandcommunity/birthsdeathsandmarriages/deaths/articles/deathsinvolvingcovid19inthecaresectorenglandandwales/deathsregisteredbetweenweekending20march2020andweekending2april2021#deaths-involving-covid-19-among-care-home-residents.

[7] Embregts Petri, Van Oorsouw Wietske, and Nijs Sara. 2020. “Impact of Infection Outbreak on Long-Term Care Staff: A Rapid Review on Psychological Well-Being.” Journal of Long-Term Care 0 (2020): 70. doi: 10.31389/JLTC.40

[8] Kisely Steve, Warren Nicola, Mcmahon Laura, Dalais Christine, Henry Irene, and Siskind Dan. n.d. “Occurrence, Prevention, and Management of the Psychological Effects of Emerging Virus Outbreaks on Healthcare Workers: Rapid Review and Meta-Analysis.” Accessed January 18, 2022a. doi: 10.1136/bmj.m1642 32371466 PMC7199468

[9] Pappa Sofia, Ntella Vasiliki, Giannakas Timoleon, Giannakoulis Vassilis G., Papoutsi Eleni, and Katsaounou Paraskevi. 2020. “Prevalence of Depression, Anxiety, and Insomnia among Healthcare Workers during the COVID-19 Pandemic: A Systematic Review and Meta-Analysis.” Brain, Behavior, and Immunity 88 (August): 901–7. doi: 10.1016/J.BBI.2020.05.026 32437915 PMC7206431

[10] Ferri Paola, Silvestri Monica, Artoni Cecilia, and Rosaria Di Lorenzo. 2016. “Workplace Violence in Different Settings and among Various Health Professionals in an Italian General Hospital: A Cross-Sectional Study.” Psychology Research and Behavior Management 9: 263. doi: 10.2147/PRBM.S114870 27729818 PMC5042196

[11] Karanikola Maria, Giannakopoulou Margarita, Mpouzika Meropi, Kaite Charis P., Tsiaousis Georgios Z., and Papathanassoglou Elizabeth D.E.. 2015. “Dysfunctional Psychological Responses among Intensive Care Unit Nurses: A Systematic Review of the Literature.” Revista Da Escola de Enfermagem Da USP 49 (5): 0847–57. doi: 10.1590/S0080-623420150000500020 26516757

[12] World Health Organization. 2011. “Psychological First Aid—Guide for Field Workers.” Who 44 (8): 813.

[13] Vernberg E.M., Steinberg A.M., Jacobs A.K., Brymer M.J., Watson P.J., Osofsky J.D., et al, 2008. Innovations in disaster mental health: Psychological first aid. Professional Psychology: Research and Practice, 39(4), p.381.

[14] Blain Daniel, Hoch Paul, and Ryan V. G.. 1945. “A Course in Psychological First Aid and Prevention.” *American Journal of Psychiatry* 101 (5): 629–34. doi: 10.1176/AJP.101.5.629

[15] Everly George S., Lating Jeffrey M., Sherman Martin F., and Goncher Ian. 2016. “The Potential Efficacy of Psychological First Aid on Self-Reported Anxiety and Mood: A Pilot Study.” The Journal of Nervous and Mental Disease 204 (3): 233–35. doi: 10.1097/NMD.0000000000000429 26919301

[16] Schoultz M., McGrogan C., Beattie M., Macaden L., Carolan C., Polson R., et al. (2022). Psychological first aid for workers in care and nursing homes: systematic review. BMC nursing, 21(1), 1–6. 35468786 10.1186/s12912-022-00866-6 PMC9038514

[17] Dieltjens Tessa, Moonens Inge, Koen Van Praet Emmy De Buck, and Vekerckhove Philippe. 2014. “A Systematic Literature Search on Psychological First Aid: Lack of Evidence to Develop Guidelines.” PLOS ONE 9 (12): e114714. doi: 10.1371/journal.pone.0114714 25503520 PMC4264843

[18] Fox Jeffrey H., Burkle Frederick M., Bass Judith, Pia Francesco A., Epstein Jonathan L., and Markenson David. 2012. “The Effectiveness of Psychological First Aid as a Disaster Intervention Tool: Research Analysis of Peer-Reviewed Literature From 1990–2010.” Disaster Medicine and Public Health Preparedness 6 (3): 247–52. doi: 10.1001/dmp.2012.39 23077267

[19] Spilsbury, K., Hanratty, B. and McCaughan, D., 2015. Supporting nursing in care homes. The RCN Foundation Patient Care and Professional Development for Nursing Staff in Care and Nursing Homes: A Research and Consultation Project.

[20] Chesney Margaret A., Neilands Torsten B., Chambers Donald B., Taylor Jonelle M., and Folkman Susan. 2006. “A Validity and Reliability Study of the Coping Self-Efficacy Scale.” *British Journal of Health Psychology* 11 (3): 421–37. doi: 10.1348/135910705X53155 16870053 PMC1602207

[21] Cohen Sheldon, Kamarck Tom, and Mermelstein Robin. 1983. “Perceived Stress Scale (PSS).” *J Health Soc Beh* 24: 285. 6668417

[22] Braun Virginia, and Clarke Victoria. 2006. “Using Thematic Analysis in Psychology.” Qualitative Research in Psychology 3 (2): 77–101. doi: 10.1191/1478088706qp063oa 32100154

[23] Scottish Care (2019) Qualifying Care; An Exploration of Social Care Registration Qualifications in Scotland.

[24] Cooper E., Spilsbury K., McCaughan D., Thompson C., Butterworth T. and Hanratty B., 2017. Priorities for the professional development of registered nurses in nursing homes: a Delphi study. Age and Ageing, 46(1), pp.39–45. doi: 10.1093/ageing/afw160 28181630

[25] Quality Compliance Systems (2022) Mandatory and Core training; what social care providers needs to know. Mandatory and Core Training: What social care providers need to know–Adult Social Care | QCS Blog.

[26] King’s Fund 2013 “Overview of the Health and Social Care Workforce | The King’s Fund.” n.d. Accessed February 11, 2022. https://www.kingsfund.org.uk/projects/time-think-differently/trends-workforce-overview.

[27] Schäfer T. and Schwarz M.A., 2019. The meaningfulness of effect sizes in psychological research: Differences between sub-disciplines and the impact of potential biases. Frontiers in psychology, 10, p.813. doi: 10.3389/fpsyg.2019.00813 31031679 PMC6470248

