# Supplementary material for: Uptake and effects of psychological first aid training for healthcare workers’ wellbeing in nursing homes: A UK national survey
Source: PLoS One. 2022 Nov 3;17(11):e0277062. doi: 10.1371/journal.pone.0277062 (PMC9632887; doi:10.1371/journal.pone.0277062)
Supplement: S1 File — (DOCX) [file pone.0277062.s001.docx]

**Supplementary Materials**

1. What are your thoughts/experiences about the Psychological First Aid (PFA) training?
2. How easy was it to access PFA training? What was your motivation for accessing/completing it?
3. What kind of support (if any) did you received regarding completion of PFA training?
4. Please describe how the PFA training influenced your experiences of working in a care/nursing home during the covid-19 pandemic?
5. How did you feel working during the pandemic? Was there difference of work experience before the PFA training and after? If so, what was that difference.
6. How easy/difficult was the PFA training?
7. What would you change about the PFA training in terms of course or timing?
8. Is PFA useful or not to help with independent work? Why?
9. How do you think PFA has helped or hindered in terms of teamwork?
10. How do you think PFA has helped/hindered with relationships outside of work?
11. How do you think PFA can be useful when faced with traumatic experiences?
12. How has PFA helped/hindered with coping during the pandemic?
13. What was the most helpful aspect of PFA for you?
14. What was the least helpful aspect of PFA for you?
15. Is there anything else you want to share with us about you experience of PFA and its usefulness/drawbacks for people working in a care/nursing home during the covid-19 pandemic?
